# An Online Multimodal Food Data Exploration Platform for Specific Population Health: Development Study

**DOI:** 10.2196/55088

**Published:** 2024-11-15

**Authors:** Lin Yang, Zhen Guo, Xiaowei Xu, Hongyu Kang, Jianqiang Lai, Jiao Li

**Affiliations:** 1 Institute of Medical Information and Library Chinese Academy of Medical Sciences/Peking Union Medical College Beijing China; 2 Key Laboratory of Medical Information Intelligent Technology Chinese Academy of Medical Sciences Beijing China; 3 Department of Biomedical Engineering School of Medical Technology Beijing Institute of Technology Beijing China; 4 National Institute for Nutrition and Health Chinese Center for Disease Control and Prevention Beijing China

**Keywords:** Chinese food data, multimodal knowledge graph, online platform, population health promotion, health promotion, nutrients, diet, pregnant women

## Abstract

**Background:**

Nutrient needs vary over the lifespan. Improving knowledge of both population groups and care providers can help with healthier food choices, thereby promoting population health and preventing diseases. Providing evidence-based food knowledge online is credible, low cost, and easily accessible.

**Objective:**

This study aimed to develop an online multimodal food data exploration platform for easy access to evidence-based diet- and nutrition-related data.

**Methods:**

We developed an online platform named Food Atlas in collaboration with a multidisciplinary expert group from the National Institute for Nutrition and Health and Peking Union Medical College Hospital in China. To demonstrate its feasibility for Chinese food for pregnant women, a user-friendly and high-quality multimodal food knowledge graph was constructed, and various interactions with graph-structured data were developed for easy access, including graph-based interactive visualizations, natural language retrieval, and image-text retrieval. Subsequently, we evaluated Food Atlas from both the system perspective and the user perspective.

**Results:**

The constructed multimodal food knowledge graph contained a total of 2011 entities, 10,410 triplets, and 23,497 images. Its schema consisted of 11 entity types and 26 types of semantic relations. Compared with 5 other online dietary platforms (Foodwake, Boohee, Xiachufang, Allrecipes, and Yummly), Food Atlas offers a distinct and comprehensive set of data content and system functions desired by target populations. Meanwhile, a total of 28 participants representing 4 different user groups were recruited to evaluate its usability: preparing for pregnancy (n=8), pregnant (n=12), clinicians (n=5), and dietitians (n=3). The mean System Usability Scale index of our platform was 82.5 (SD 9.94; range 40.0-82.5). This above-average usability score and the use cases indicated that Food Atlas is tailored to the needs of the target users. Furthermore, 96% (27/28) of the participants stated that the platform had high consistency, illustrating the necessity and effectiveness of health professionals participating in online, evidence-based resource development.

**Conclusions:**

This study demonstrates the development of an online multimodal food data exploration platform and its ability to meet the rising demand for accessible, credible, and appropriate evidence-based online dietary resources. Further research and broader implementation of such platforms have the potential to popularize knowledge, thereby helping populations at different life stages make healthier food choices.

## Introduction

The relationship between diet and health at each life stage has been extensively investigated [1-5]. Using these findings to help specific populations establish healthy dietary patterns has a beneficial impact on health promotion and disease prevention [6,7]. To promote well-being for all at all ages, many countries publish separate food-based dietary guidelines for subpopulations, including infants, school-age children, adolescents, pregnant and lactating women, older adults, and others [8]. However, adherence to food-based dietary guidelines is low [9-11]. Unhealthy diets are believed to be responsible for 1 in every 5 deaths globally [12]. A key challenge for population groups to adhere to dietary recommendations is having inadequate knowledge of dietary recommendations and receiving limited information from their care providers [13,14].

Improving knowledge is in fact a first step and aims to help different populations make healthier food choices or change their current eating habits [15]. Previous studies have shown that evidence-based, online educational interventions should be credible, low cost, and easily accessible [16,17]. It is important to popularize internet-based education and knowledge about a healthy diet for populations at every life stage.

However, this problem has not been well addressed so far [18,19]. Much health-related dietary advice online is inaccurate [20,21]. Whether nutritional content is intended for a specific population is not clearly stated [22]. According to the meta-analysis by Zhang and Kim [23], users of online health information largely rely on peripheral cues (such as system navigability and aesthetics) and lack the knowledge and skills to evaluate the quality of online health information. Credible online food data sets like food composition databases, which contain detailed information on the nutritional composition of foods and other relevant compounds (eg, phytochemicals, antinutrients, bioactive compounds, toxic compounds), are critical for estimations in relation to nutrition and public health, as well as for different calculations in food science [24-26]. However, they are neither easy for the public to use nor sufficiently linked to human health, missing some important food properties such as food form and degree of processing [27]. Online recipe sites often share recipes and cooking tips for kitchen experts and home cooks [28,29]. People can discover recipes, personalize them, and make food choices through recipe photos, videos, ratings, comments, and bookmarks. However, these sites often show popular recipes that are not always the healthiest options and can promote an unhealthy lifestyle [30,31]. This highlights a pressing need to help different population groups more easily find the evidence-based diet- and nutrition-related data they want or need, which requires integrating distributed reliable data and providing user-friendly data access.

We aimed to develop an online food data exploration platform to provide easily accessible, credible, and appropriate evidence-based online food knowledge to help populations at different life stages make healthier food choices, as evidenced in previous studies [23]. In the food industry, abundant multimodal data, such as images and videos, exist. These visual descriptions offer comprehensive food information that supports users in making informed and health-conscious selections [32]. Since they usually have intrinsic semantic associations, we considered that the graphical representation of such multimodal data may be helpful for better discovery and utilization. Previous studies have shown that knowledge graphs, which are multirelational graphs of data with nodes representing entities and edges representing different types of relations [33], can effectively organize data and represent knowledge in the field of food science and industry [34,35]. A multimodal food knowledge graph can help food-oriented multimodal learning technologies support many cross-modal tasks, such as cross-modal recipe-food image retrieval [36] and recipe recommendations [37]. Moreover, explore, perceive, and reason with graph-structured data can facilitate the understanding and consumption of information. Bellmann et al [38] proposed attribute association graphs to enable interpretability and intuitive visual medical data exploration. However, most existing food knowledge graphs focus on organizing verbal knowledge, regardless of visual data. Though there have been some initial attempts to incorporate visual information into knowledge graphs [39,40], these graphs are not available to the public for navigation online. Compared with previous studies, we focused on graphical representation of multimodal food data curated from evidence-based resources and how to interact with graph-structured data, aiming to facilitate information seeking.

## Methods

### Food Atlas Workflow

**Figure 1:**
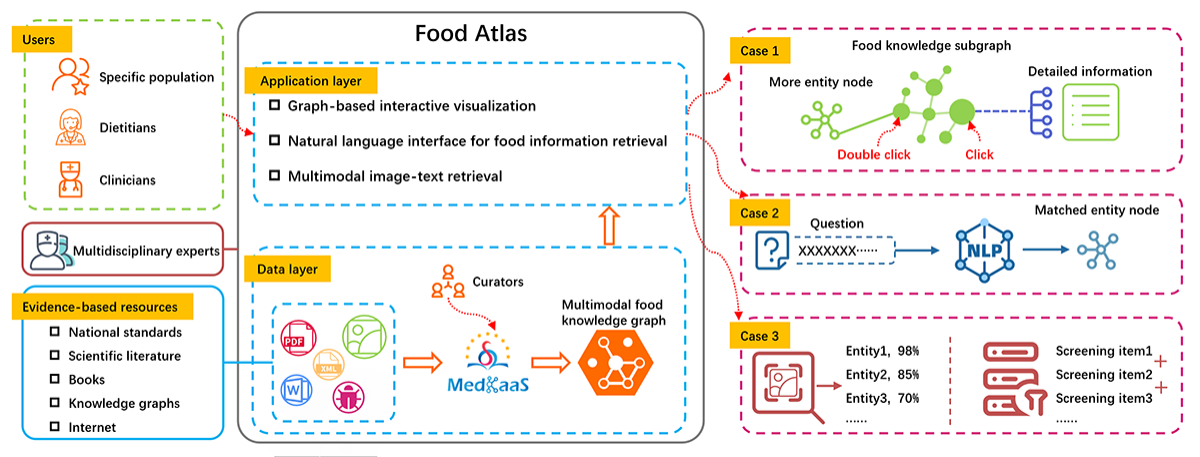
Workflow for Food Atlas construction. NLP: natural language processing.

To provide easily accessible, evidenced-based food knowledge online, we developed a platform named Food Atlas that could support diversified food information retrieval for different user groups, including specific populations, clinicians, and dietitians. The workflow of its construction is shown in Figure 1. The details are described in the following paragraphs.

### Information Needs Analysis at the Population Level

To clarify the information needs at the population level, we collaborated with experts from the National Institute for Nutrition and Health, Chinese Center for Disease Control and Prevention, and clinicians and dietitians from Peking Union Medical College Hospital to understand the characteristics and needs of different populations. Combined with previous diet-related surveys [41], we summarized the following key insights: (1) There was a need for evidence-based dietary information to address issues such as low data quality and data inconsistency; (2) dietary recommendations are needed to alleviate physical symptoms that impact the individual experience of nutrition-related actions (such as fatigue, physical discomfort, food aversions , nausea, and complications); (3) practical information is needed to help put nutritional guidelines into practice.

Meanwhile, food choices are influenced by a diversity of factors that interact among them to produce a final behavior. Variability in food characteristics, including food constituents and technological processing, as well as variations in population groups’ characteristics, such as nutrient needs and physiology, can greatly affect the final decision [42,43]. Based on the corresponding factors, as well as important aspects with a close connection to the topic of food and population health, we collectively identified the core data content (such as ingredients, nutrients, cooking method, and dietary function) that needed to be covered in our multimodal food knowledge graph. We then analyzed the characteristics of information retrieval inputs for specific populations, clinicians, and dietitians, as well as based on our experience from a previous study [44]. After several rounds of discussion, we determined the system functions, including graph-based interactive visualizations, natural language retrieval, and image-text retrieval.

### Multimodal Food Knowledge Graph Construction

According to the core data content identified, our food-oriented graph consisted of triples, defined as T = (E, R, E), where E represented entities and R represented relations. Its schema mainly contains 11 entity types of verbal knowledge (including food, food category, ingredients, population, synonym name, dietary function, nutrients, cooking method, cookware, region, and season), as well as visual knowledge (visual perceptions reflected by images). We chose pregnant women as our target population and demonstrated how we used MedKaaS [45] to construct a multimodal Chinese food knowledge graph from evidence-based resources. MedKaaS is a tool set we developed for medical knowledge processing that is equipped with the knowledge schema design tool, knowledge extractor tool, knowledge fusion tool, and quality control tool.

First, a multidisciplinary expert group was established to guide the collection and selection of evidence-based resources. This group included obstetricians, nutritionists, dietitians, pediatricians, and Chinese medicine practitioners, all possessing extensive expertise with over 20 years of experience in prenatal nutrition. We then conducted searches for literature and books from PubMed and CNKI, as well as websites such as Amazon and JD, using predefined keywords including “(‘home-cooked dishes’ OR ‘recipe’) AND ‘pregnant woman’,” “‘pregnancy’ AND (‘nutrition’ OR ‘diet’ OR ‘dietary pattern’).” After manually reviewing the retrieved results, we removed irrelevant publications. Considering that there were few studies on whether specific Chinese foods were beneficial to maternal and infant health, we selected recipe books written by clinical nutritionists from third-class hospitals in China as the primary resources for foods in our knowledge graph. Subsequently, we collaborated with the multidisciplinary expert group to identify crucial nutrients and their associations with maternal and infant health from the retrieved literature. To unify category, we mainly referred to the Food Production License Classification Catalogue, 2017 Classification of National Economic Industries (GBT 4754-2017), and dietary patterns in the literature to define our food categories. Meanwhile, we integrated relevant content from existing food knowledge graphs [35,46]. After that, we used the knowledge graph schema design tool of MedKaaS to establish 11 classes and corresponding relations. A team of 3 annotators was then recruited. All annotators had a dietary research background and annotating experience. Two of the annotators used the knowledge extractor tool to curate all entities and their relations independently for each qualified food from the selected resources. This tool combines large language models and machine learning algorithms to ensure the effective extraction of evidence-based knowledge. Since the quality control tool showed that the consistency rate of annotation was 92%, a senior third-party annotator resolved disagreements. For images corresponding to these entities, we defined a set of image collection rules, including resolution, format, and content, and curated them through 2 search engines, Baidu and Bing. To ensure that the Food Atlas remained up to date, we regularly collected and curated incremental publications through the knowledge fusion tool. An overview of the data sources in Food Atlas is shown in Table S1 in Multimedia Appendix 1. The curation results were finally reviewed by the multidisciplinary expert group. Thereby, this knowledge graph contained a total of 2011 entities, 10,410 triplets, and 23,497 images.

### Development of the Multimodal Food Data Exploration Platform

To meet the needs of user information retrieval, Food Atlas mainly supported graph-based interactive visualizations, natural language retrieval, and image-text retrieval.

#### Graph-Based Interactive Visualization

Visual learning is one of the primary forms of interpreting information. Interactive visualizations play a key part in the understanding and exploration of data. It can inspire visual thinking and increase motivation for learning in an easy-to-use interface [47,48]. Previous studies showed that interactive visualizations of semantic search results could be more effective at helping users query ontologically structured knowledge to find and understand the information needed [49]. Accordingly, we decided to use graph- and network-based interactive visualization techniques to present our multimodal food knowledge graph.

We used a Neo4j [50] graph database to store graph-based food data and execute semantic queries via Cypher [51], a graph query language designed specifically for Neo4j. The query results are organized as a knowledge graph with g6-powered [52] interactive visualizations. Since memorability is important in the presentation [53] and colorful visualizations result in higher memory scores, with 7 or more colors being the best [54,55], we color-coded each node based on its entity category. To make the view interesting and vivid, we presented the corresponding image (if applicable) in the node. Moreover, several convenient functions were built to help interact and explore with the knowledge graph, including (1) node expansion: when a user double clicks on an entity node, the system will expand the node and show the subgraph with all other related nodes and the relations and (2) node information box on the right sidebar: when a node is selected, more information about the node such as the entity type and name of an ingredient is shown on the right side of the screen.

#### Natural Language Interface for Food Information Retrieval

People are usually familiar with using natural language to express their information needs: for example, “what foods are rich in folic acid?” It is not easy for them to write a question in a formal query language (eg, Cypher). Accordingly, natural language interfaces are proposed to improve the usability of a retrieval system [56,57]. It allows end users to access information stored in databases by typing requests expressed in natural language (eg, Chinese and English). Compared with a graphical interface, it requires less prior knowledge about system functionality and use details to work with it [58]. Therefore, we decided to equip Food Atlas with a natural language interface. The key problem is how to translate free-text inputs to executable graph database queries.

**Figure 2:**
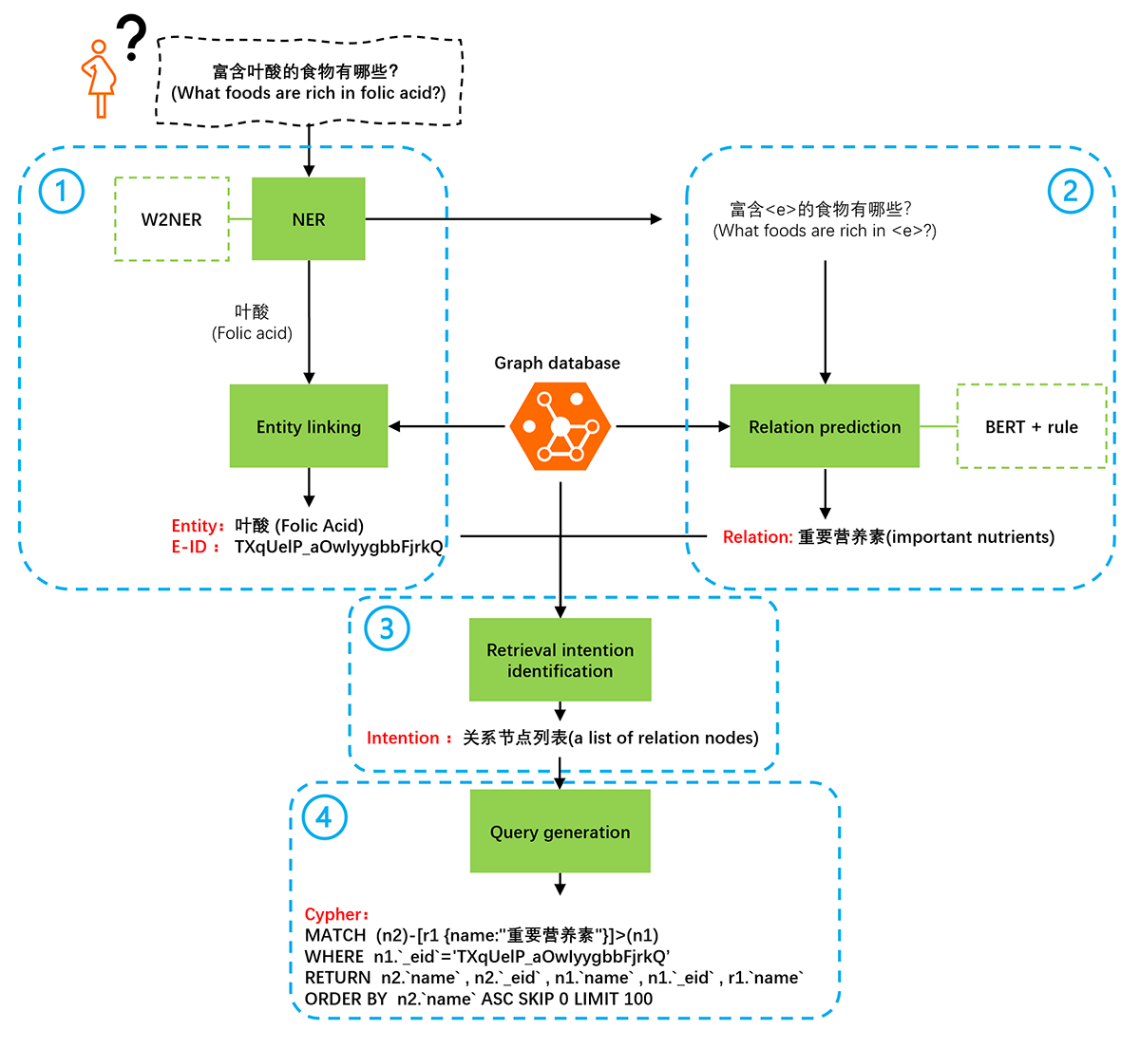
Workflow to translate natural language into a graph database query. BERT: Bidirectional Encoder Representations from Transformers; NER: named entity recognition.

Since the graph consisted of a set of “entity-relation-entity,” we tried to transfer the retrieval problem into a search for related entities when given an entity and its relation. Referring to recent work [59,60], we used a 4-step method to generate graph query statements from natural language (Figure 2). First, we used a named entity recognition method called W2NER (named entity recognition [NER] as word-word relation classification) [61] to identify entities in a given free-text question. Each entity was then linked to the corresponding one in the knowledge graph. Second, we took the question with an entity removed as input and predicted the relation using a classifier combining Bidirectional Encoder Representations from Transformers (BERT) [62] and rules. Third, we identified the potential retrieval intention based on both the original question and the extracted text. Fourth, a graph database query was formulated with predefined rules. Finally, the semantic search results were organized into a subgraph and fed back to the end user.

Moreover, we integrated the machine translation application programming interface (API) [63] into our natural language interface, facilitating use by native English speakers.

#### Multimodal Image-Text Retrieval

In addition to querying via natural language, image-text search can be a powerful addition for food information retrieval. In our scenario, such multimodal retrieval uses a query represented by an image to retrieve other texts or images in the graph database. The key challenge is to eliminate the heterogeneous gap between different patterns.

Existing mainstream methods primarily focus on modeling the association of image-text pairs. Benefiting from the accessibility of massive image-text pairs from the web, large-scale vision-language pretraining frameworks can extract multimodal representations in a unified form and achieve promising performance when transferred to downstream tasks [64-66]. Compared with other methods [67], Contrastive Language-Image Pre-training (CLIP) [68] has emerged as a renowned method to train vision encoders to generate image and text representations, facilitating various applications. Recently, CLIP has become the default choice for the vision backbone for multimodal large language models [69] to connect image inputs for language interactions. Considering its superior prior knowledge in aligning vision and language [70], it has the potential to effectively support our image-text retrieval task. To achieve multimodal food information retrieval, we first converted the food image query to a CLIP embedding. Similar image-text pairs in our graph database were then identified using cosine similarity. After that, the top 5 most similar food names were output. The end user can select one of the names for detailed information.

### Technical Specification Certification and Usability Assessment

We evaluated Food Atlas from both the system perspective and the user perspective. From the system perspective, our platform complies with “CESI/TS 021-2020: Certification techniques specifications for knowledge graph construction platform” and “CESI/TS 043-2022: Certification techniques specifications for medical knowledge graph construction platform” issued by the China Electronics Standardization Institute and has obtained certifications. Additionally, we selected 5 representative online dietary platforms for functional comparison.

From the user perspective, we conducted a usability evaluation. We recruited participants from midwifery institutions in Beijing, China, including women preparing for pregnancy (or their spouses), pregnant women (or their spouses), clinicians, and dietitians. Participants were invited to try out Food Atlas, and the System Usability Scale (SUS) questionnaire [71] was used to conduct small-scale user surveys. This questionnaire has a 5-point Likert scale, ranging from “Strongly Agree” to “Strongly Disagree,” for 10 items. A total score of 68 (or higher) is regarded as “above average usability” [71]. An online survey platform, WJX, was used to collect survey data, and R (version 4.4.0; The R Foundation) was used for statistical analysis.

### Ethical Considerations

This study was approved by the Ethics Committee of the Institute of Medical Information, Chinese Academy of Medical Sciences (IMICAMS/01/21/HREC). All participants were informed that their responses would be used to inform public-facing research. All procedures were performed in accordance with the Declaration of Helsinki.

## Results

### Overview of the Multimodal Food Knowledge Graph

**Figure 3:**
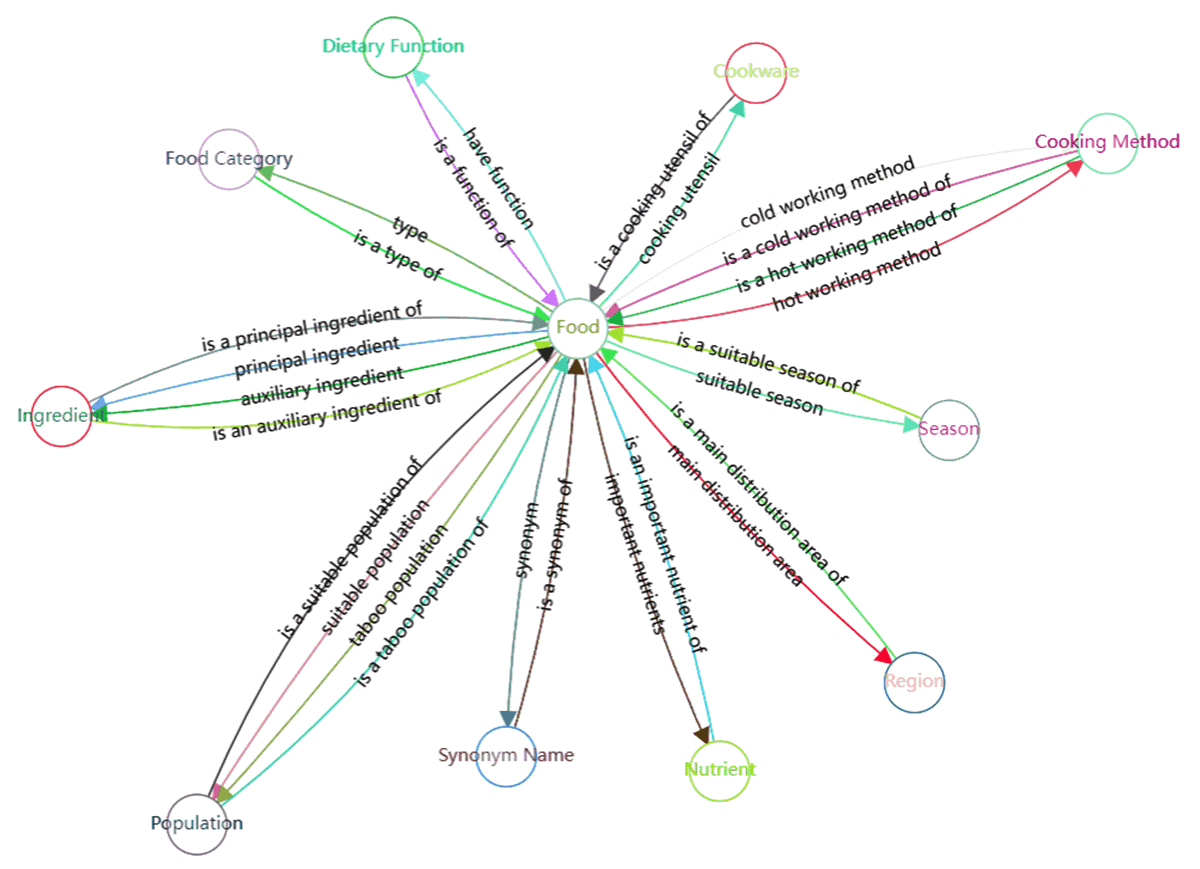
Schema of the multimodal food knowledge graph.

The schema of our multimodal food knowledge graph consists of 26 types of semantic relations among 11 entity types. Figure 3 details the correspondence between entities and relations.

The constructed food knowledge graph contains a total of 2011 entities, 10,410 triplets, and 23,497 images. Table 1 lists the descriptive statistics of our multimodal food knowledge graph.

**Table 1:** Descriptive statistics of the multimodal food knowledge graph: entity count per type, triplet count per semantic relation, and image count.

Knowledge graph		Results, n
Entities per type (n=2011)		
	Food	253
	Synonym name	1090
	Ingredient	352
	Nutrient	38
	Food category	4
	Population	1
	Dietary function	196
	Cookware	37
	Cooking method	23
	Season	4
	Region	13
Triplets per semantic relation (n=10,410)		
	<Food, type, food category>	253
	<Food category, is a type of, food>	253
	<Food, synonym, synonym name>	1090
	<Synonym name, is a synonym of, food>	1090
	<Food, principal ingredient, ingredient>	784
	<Ingredient, is a principal ingredient of, food>	784
	<Food, auxiliary ingredient, ingredient>	800
	<Ingredient, is an auxiliary ingredient of, food>	800
	<Food, suitable population, population>	253
	<Population, is a suitable population of, food>	253
	<Food, taboo population, population>	0
	<Population, is a taboo population of, food>	0
	<Food, have function, dietary function>	253
	<Dietary Function, is a function of, food>	253
	<Food, important nutrient, nutrient>	537
	<Nutrient, is an important nutrient of, food>	537
	<Food, cold working method, cooking method>	165
	<Cooking method, is a cold working method of, food>	165
	<Food, hot working method, cooking method>	328
	<Cooking method, is a hot working method of, food>	328
	<Food, cooking utensil, cookware>	236
	<Cookware, is a cooking utensil of, food>	236
	<Food, main distribution area, region>	253
	<Region, is a main distribution area of, food>	253
	<Food, suitable season, season>	253
	<Season, is a suitable season of, food>	253
Images		23,497

### Overview of Food Atlas

#### Use of Food Atlas

**Figure 4:**
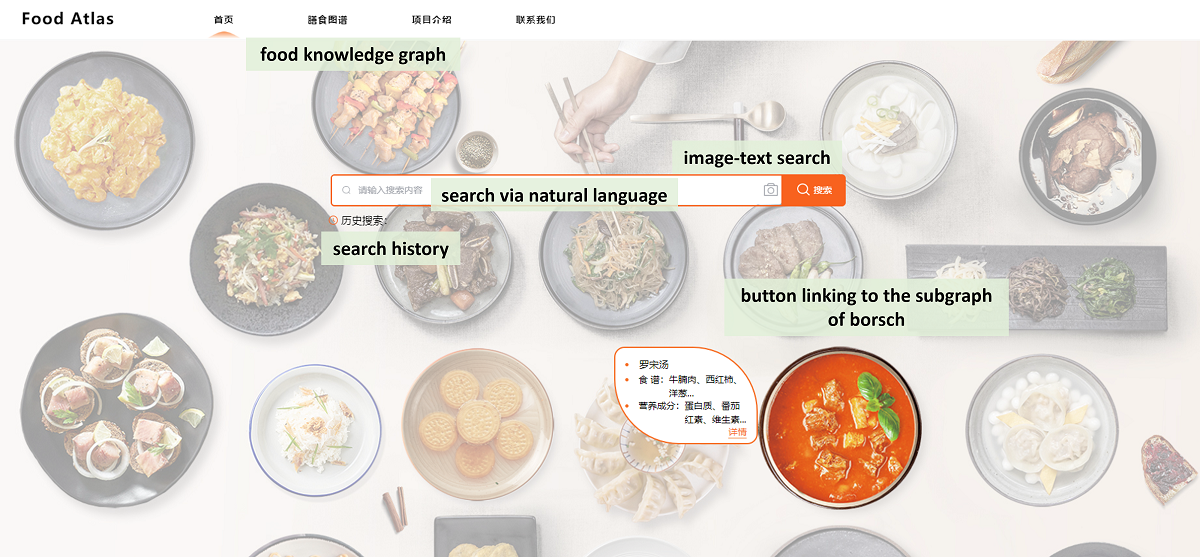
Home page of Food Atlas.

In order to provide various interactions with graph-structured data to facilitate access by different user groups, Food Atlas mainly supports graph-based interactive visualizations, natural language retrieval, advanced search, and image-text retrieval. Figure 4 shows its home page [72]. This page consists of 3 parts: (1) a navigation bar to link to appropriate sections and pages of Food Atlas, (2) a search box for users to enter free-text queries or images, (3) buttons to link to specific subgraphs by food.

#### Functional Comparison

We selected 5 representative online dietary platform, including Foodwake [73], Boohee [74], Xiachufang [75], Allrecipes [28], and Yummly [29], and compared them with Food Atlas in terms of data content and system functions desired by the target populations (Table 2). The results showed that one of the advantages of Food Atlas is its comprehensive method of providing dietary information.

**Table 2:** Functional comparison between Food Atlas and 5 other online dietary platforms.

Functional module	Online dietary platform					
	Food Atlas	Foodwake	Boohee	Xiachufang	Allrecipes	Yummly
Keyword search	X^a^	X	X	X	X	X
Q&A^b^	X	X	—^c^	X	—	—
Image-text search	X	—	—	—	—	—
Knowledge graph	X	X	—	—	—	—
Ingredients	X	—	X	X	X	X
Cooking method	X	—	X	X	X	X
Nutrients	X	X	X	X	X	X
Dietary function	X	X	—	X	—	—

^a^Has the indicated function.

^b^Q&A: question and answer.

^c^Does not have the indicated function.

#### Use Cases

**Figure 5:**
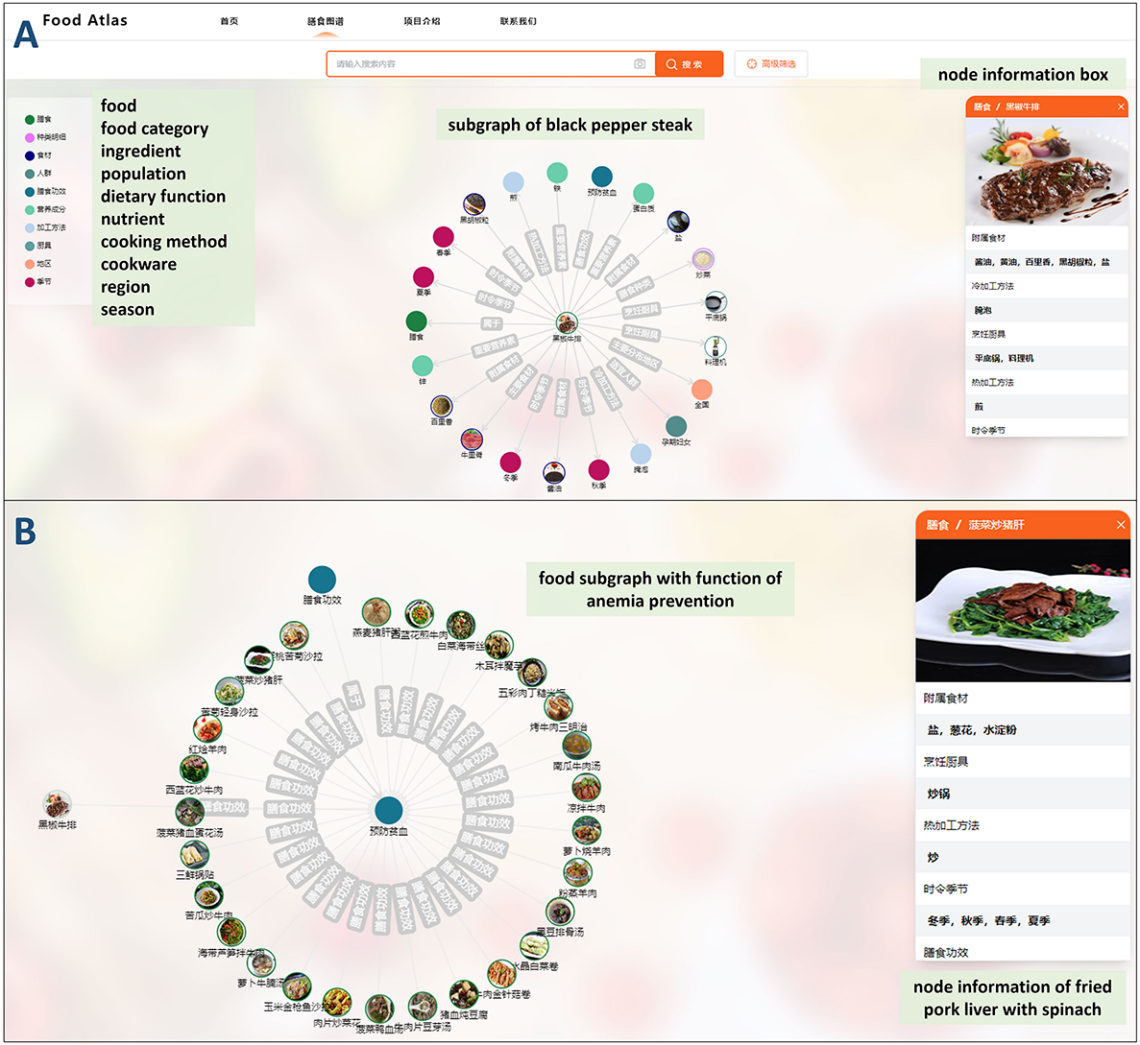
Interactive visualization of the multimodal food knowledge graph, including (A) the default food knowledge graph page and (B) a subgraph that appears after double-clicking an entity node.

Food Atlas allows users to interactively explore the multimodal food knowledge graph. Assuming a pregnant woman is not familiar with the system, she randomly selects “膳食图谱” (food knowledge graph) from the navigation bar. The system will provide the detailed information of the default food (eg, black pepper steak) in the form of a graph (Figure 5A). She may find this dish helpful for anemia prevention and want to know what other dishes have the same function. She can double-click the entity node to learn more (Figure 5B).

**Figure 6:**
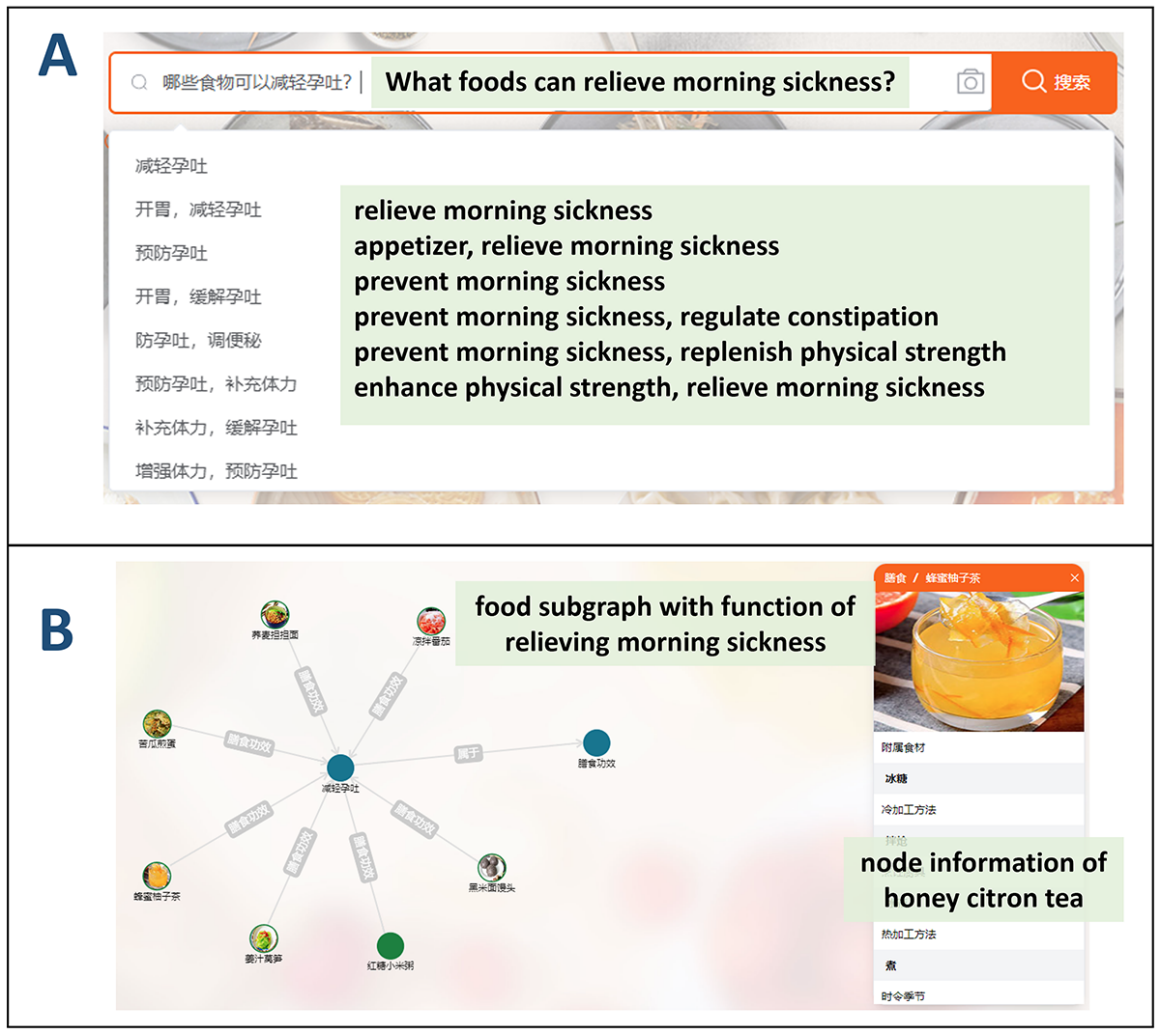
Natural language interface for food information seeking, including (A) entities identified from the query and (B) a subgraph that appears after selecting an identified entity.

For a clinician, he may often be asked what food can relieve morning sickness. He can use our natural language interface to input the query “哪些食物可以减轻孕吐?” (What foods can relieve morning sickness?). Food Atlas can successfully identify related dietary functions (eg, “减轻孕吐” [relieve morning sickness]) and provide the list below the search box (Figure 6A). Once the clinician selects it, the system will return all food entities that contain this function as a graph (Figure 6B).

**Figure 7:**
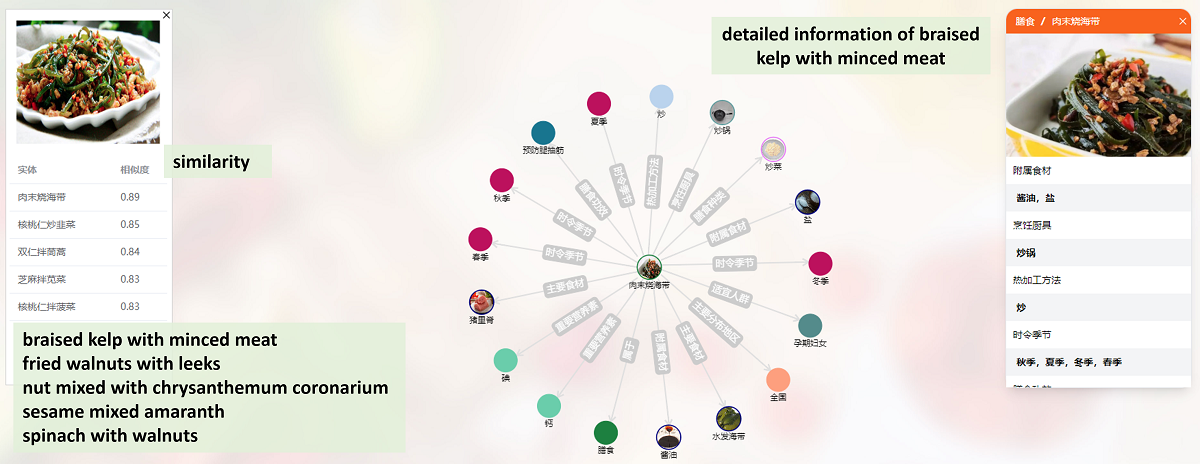
Image-text retrieval by Food Atlas, in which the top 5 most similar foods are identified and a subgraph is provided for the top-ranked food.

Suppose a pregnant woman wants to know if a dish she saw on social media is suitable for her. She can upload the image of the dish (eg, braised kelp with minced meat), and Food Atlas will list the top 5 most similar foods as well as their similarity values (Figure 7). She will find that the first one is the dish she is looking for, learn more information, and make a decision.

**Figure 8:**
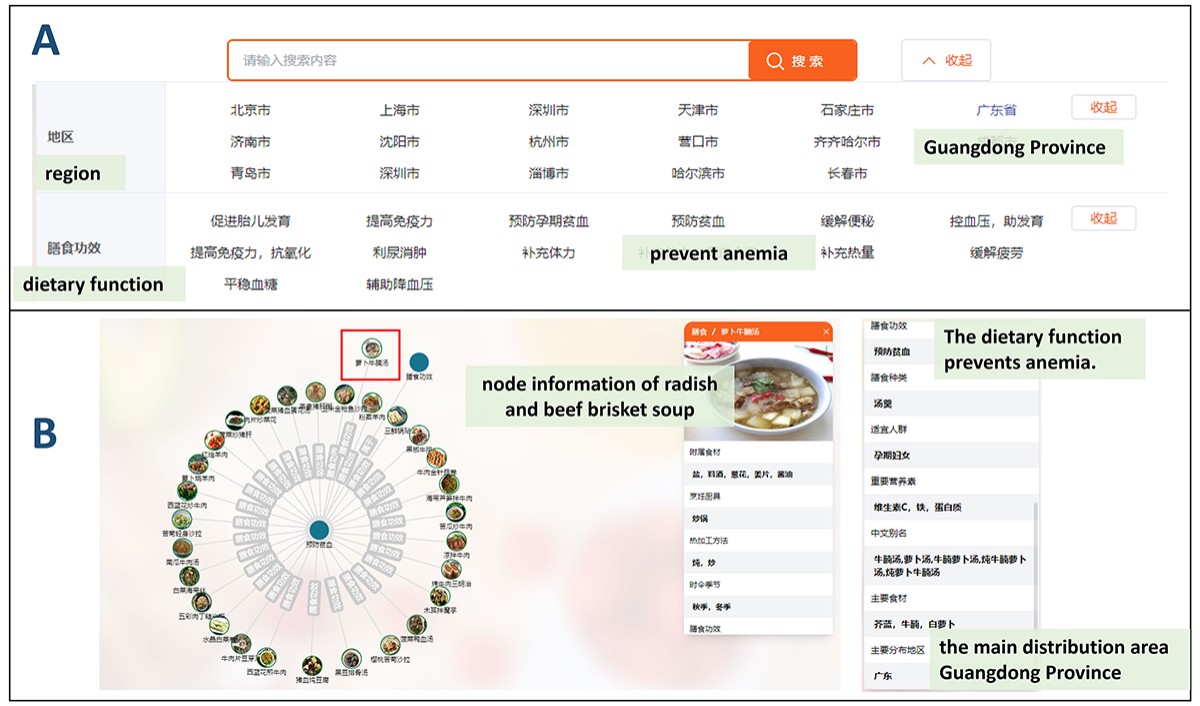
Composite information retrieval by Food Atlas, including (A) the advanced search page and (B) the page for the food that meets the filter conditions.

If a dietitian needs to give dietary advice to prevent anemia for a pregnant woman from Guangdong Province, he can use the advanced search to formulate a complex query. Dietary function and region are selected as screening items, while anemia prevention and Guangdong Province are selected as screening conditions (Figure 8A). Food Atlas can find that “萝卜牛腩汤” (radish and beef brisket soup) meets her nutrient needs and dietary habits (Figure 8B).

### Assessment of Food Atlas Usability

A total of 28 participants used Food Atlas and evaluated its usability. Their demographic characteristics are shown in Table 3. The results showed that the target users of our platform were relatively broad, covering specific populations and their family members. One-half (14/28, 50%) of the participants (including all 3 dietitians) had experience using similar platforms, although the proportion was lower in the “preparing for pregnancy” and “pregnancy” groups (7/28, 35%).

**Table 3:** Demographics of participants in 4 groups (N=28): preparing for pregnancy, pregnancy, clinicians, and dietitians.

Demographics			Preparing for pregnancy (n=8)		Pregnancy (n=12)		Clinicians (n=5)		Dietitians (n=3)
Age (years), mean (SD)			28.63 (3.66)		30.17 (2.76)		45.4 (4.51)		44.33 (6.66)
Age group (years), n (%)									
	<35	7 (88)		11 (92)		0 (0)		0 (0)	
	≥35	1 (13)		1 (8)		5 (100)		3 (100)	
Gender, n (%)									
	Male	3 (38)		3 (25)		1 (20)		2 (67)	
	Female	5 (63)		9 (75)		4 (80)		1 (33)	
Education level, n (%)									
	Associate degree or below	2 (25)		2 (17)		0 (0)		0 (0)	
	Bachelor’s degree	3 (38)		6 (50)		0 (0)		1 (33)	
	Master’s degree or above	3 (38)		4 (33)		5 (100)		2 (67)	
Pregnancy experience, n (%)									
	Yes	1 (13)		2 (17)		5 (100)		3 (100)	
	No	7 (88)		10 (83)		0 (0)		0 (0)	
Similar platform use experience, n (%)									
	Yes	3 (38)		4 (33)		4 (80)		3 (100)	
	No	5 (63)		8 (67)		1 (20)		0 (0)	

The mean SUS index was 82.5 (SD 9.94; range 40.0-82.5), which indicates an above-average usability score. Table 4 displays the results of the SUS questionnaire. A total of 93% (26/28) of the participants wanted to use Food Atlas frequently, and 96% (27/28) stated that the platform had high consistency. This showed that Food Atlas was tailored to the needs of the target users, and its underlying data were reliable.

**Table 4:** System Usability Scale (SUS) questionnaire scores for Food Atlas (N=28).

Statements	Disagree^a^, n (%)	Neutral, n (%)	Agree^b^, n (%)
I think that I would like to use the platform frequently.	0 (0)	2 (7)	26 (93)
I found the platform unnecessarily complex.	16 (57)	5 (18)	7 (25)
I thought the platform was easy to use.	5 (18)	9 (32)	14 (50)
I think that I would need assistance to be able to use the platform.	17 (61)	8 (29)	3 (11)
I found the various functions in the platform were well integrated.	5 (18)	3 (11)	20 (71)
I thought there was too much inconsistency in the platform.	27 (96)	1 (4)	0 (0)
I would imagine that most people would learn to use the platform very quickly.	6 (21)	3 (11)	19 (68)
I found the platform very cumbersome to use.	14 (50)	8 (29)	6 (21)
I felt very confident using the platform.	6 (21)	7 (25)	15 (54)
I needed to learn a lot of things before I could get going with the platform.	21 (75)	4 (14)	3 (11)

^a^Scores 1 and 2 were combined and clustered under the heading of “Disagree.”

^b^Scores 4 and 5 were combined and clustered under the heading of “Agree.”

## Discussion

### Principal Findings

In this study, we collaboratively developed an online food data exploration platform, Food Atlas, with a multidisciplinary expert group. To demonstrate its feasibility for Chinese food for pregnant women, a user-friendly and high-quality food knowledge graph was provided; it included 2011 entities, 10,410 triplets, and 23,497 images, as well as diversified food information retrieval for different user groups. Our previous study [16] showed that an online prenatal education curriculum focusing on nutrition has the potential to reduce adverse outcomes in pregnant women. Considering information-seeking behaviors, perspectives, and preferences, multimodal resources are needed to reach people and enhance engagement with evidence-based information and health care [32,44,76]. Therefore, there is an urgent need for an easily accessible, credible, and appropriate evidence-based online food platform to promote healthy diets in specific populations, especially in this digital era.

To guarantee the high quality of Food Atas, we selected, assessed, and curated evidence-based dietary resources and established an update mechanism. It is difficult to determine whether a food, especially a mixed dish, is suitable for a certain population. This requires identifying reliable data sources, combining food information with healthy dietary patterns or recommendations for different populations, and coordinating inconsistencies among different data sources. In particular, the effects of some ingredients or compounds (such as alternative sweeteners) on human health are still unclear [77]. To address this issue, we worked closely with experts from clinical and nutrition-related institutions. Their extensive expertise helped us quickly identify high-quality evidence and standardize our food knowledge curation workflow. Of the participants, 96% (26/28) stated that the platform had high consistency, illustrating the necessity and effectiveness of health professionals participating in online evidence-based resource development.

We graphically represented curated multimodal food data, aiming to optimize the organization, presentation, and interaction of knowledge. A graph is a type of sparse data structure that consists of nodes and edges. Compared with other schemas, it can show knowledge more comprehensively, especially the relations between knowledge nodes [34]. Platforms like Foodwake also offer support for the graphical representation of food knowledge, which in turn shows its necessity. Different from existing food knowledge graphs about recipes [78], food safety [79], nutrient, and health [80], the schema we proposed covers the key content for a healthy diet for specific populations and has the ability to support the description of Chinese food. The constructed knowledge graph includes what the food is, what the dish is, when to eat, where to eat, how to cook, and who can eat. Both verbal knowledge and visual data are involved. Meanwhile, its construction process complied with 2 technical specifications issued by the China Electronics Standardization Institute and has been certified.

Furthermore, we offered various interactions with graph-structured data for easy access, as evidenced in previous studies [81]. Users can choose the appropriate interface according to their personal preferences or information needs, rather than being forced into a limited mode of communication. We used specific use cases to illustrate its “fitness of purpose” for various downstream applications, which has been carried out in previous related work [82,83]. Compared with other online dietary platforms, Food Atlas offers a distinct set of functions that can meet the specific needs of different users. An above-average usability score (82.5) indicates that Food Atlas is tailored to the needs of the target users, which also demonstrates the feasibility of our technical route.

Our online food data exploration platform, Food Atlas, serves as a valuable source of dietary knowledge, enhancing target users’ knowledge regarding nutrition at different life stages, recommended practices, and diets to alleviate physical discomfort symptoms. Given that people increasingly seek information on the web [23], our platform not only meets the rising demand for accessible, credible, and appropriate evidence-based online dietary resources but also enhances the engagement of health professionals. By continually updating and promoting this platform more widely, we aim to encourage healthier diets and improve health.

### Limitations

Our study has several limitations. First, it is a feasibility study based on Chinese food to promote health for pregnant women. The small-scale data sets limit its real impact. In addition, whether Food Atlas can be applied to other cuisines (such as western food and Japanese food) and population groups and to what extent it needs to be localized still need to be clarified and validated. Second, our platform lacks the traceability of evidence, and being equipped with this function will further enhance its reliability. Third, we did not quantitatively evaluate the performance of our natural language retrieval and image-text retrieval. In fact, existing state-of-the-art models [60,67] can be used in the corresponding system modules. With their assistance, the retrieval results would be improved. In the future, we will validate the feasibility and effectiveness of these methods.

### Conclusions

This study outlined the development of an easily accessible, credible, and appropriate evidence-based online food platform for specific population health promotion and assessed its fitness for different user groups. The results indicate the necessity and effectiveness of health professionals participating in online evidence-based resource development. Various interactions and navigation strategies of graph-structured food data can meet the information needs of different user groups. The growing prominence of online evidence-based food resources presents an opportunity for healthy diets. To optimize Food Atlas, further research should focus on the potential feasibility for other population groups and cuisine, as well as state-of-the-art models.

## Appendix

Multimedia Appendix 1 An overview of the data sources for the multimodal food knowledge graph.

## Abbreviations

API: application programming interface
BERT: Bidirectional Encoder Representations from Transformers
CLIP: Contrastive Language-Image Pre-training
NER: named entity recognition
SUS: system usability scale

## Data Availability

The data sets generated during this study are not publicly available due to copyright issues but are available from the corresponding author on reasonable request.
