# Supplementary material for: An Online Multimodal Food Data Exploration Platform for Specific Population Health: Development Study
Source: JMIR Form Res. 2024 Nov 15;8:e55088. doi: 10.2196/55088 (PMC11607570; doi:10.2196/55088)
Supplement: Multimedia Appendix 1 [file formative_v8i1e55088_app1.docx]

Table S1. An overview of the data sources for the multimodal food knowledge graph.

| **Resource type** | **Source** |
| --- | --- |
| National standards | Food Production Safety Supervision and Management Departmen. Food Production License Classification Catalogue. 2020. Available from: https://www.gov.cn/zhengce/zhengceku/2020-03/27/content_5496236.htm [accessed Sep 27, 2024] |
|  | State Administration for Market Regulation. Measures for the Administration of Food Production Licensing. 2020. Available from: https://www.gov.cn/gongbao/content/2020/content_5509732.htm [accessed Sep 27, 2024] |
|  | China AQSIQ, the Standardization Administration of China. Classification of National Economic Industries. Available from: https://www.stats.gov.cn/xxgk/tjbz/gjtjbz/201710/P020200612582987902992.PDF [accessed Sep 27, 2024] |
|  | Ministry of Health of the People’s Republic of China. General Rules for Nutrition Labelling of Pre-Packaged Food. 2011. Available from: http://www.nhc.gov.cn/ewebeditor/uploadfile/2013/06/20130605104041625.pdf [accessed Sep 27, 2024] |
| Scientific literature | Sánchez-García JC, Saraceno López-Palop I, Piqueras-Sola B, Cortés-Martín J, Mellado-García E, Muñóz Sánchez I, Rodríguez-Blanque R. Advancements in Nutritional Strategies for Gestational Diabetes Management: A Systematic Review of Recent Evidence. J Clin Med 2023 Dec 20;13(1):37. PMID:38202044 |
|  | Hayashi I, Sakane N, Suganuma A, Nagai N. Association of a pro-inflammatory diet and gestational diabetes mellitus with maternal anemia and hemoglobin levels during pregnancy: a prospective observational case-control study. Nutr Res 2023 Jul;115:38–46. PMID:37295325 |
|  | Ter Borg S, Koopman N, Verkaik-Kloosterman J. An Evaluation of Food and Nutrient Intake among Pregnant Women in The Netherlands: A Systematic Review. Nutrients 2023 Jul 7;15(13):3071. PMID:37447397 |
|  | Ter Borg S, Koopman N, Verkaik-Kloosterman J. An Evaluation of Food and Nutrient Intake among Pregnant Women in The Netherlands: A Systematic Review. Nutrients 2023 Jul 7;15(13):3071. PMID:37447397 |
|  | Winter HG, Rolnik DL, Mol BWJ, Torkel S, Alesi S, Mousa A, Habibi N, Silva TR, Oi Cheung T, Thien Tay C, Quinteros A, Grieger JA, Moran LJ. Can Dietary Patterns Impact Fertility Outcomes? A Systematic Review and Meta-Analysis. Nutrients 2023 May 31;15(11):2589. PMID:37299551 |
|  | Jevitt CM, Ketchum K. Pairing Evidence-Based Strategies With Motivational Interviewing to Support Optimal Nutrition and Weight Gain in Pregnancy. J Perinat Neonatal Nurs 2024 Mar 1;38(1):25–36. PMID:38278641 |
|  | Cf D, D A, Md C, Mj H, Em S, Ma Z. Nutritional and Exercise-Focused Lifestyle Interventions and Glycemic Control in Women with Diabetes in Pregnancy: A Systematic Review and Meta-Analysis of Randomized Clinical Trials. Nutrients Nutrients; 2023 Sep 1;15(2). PMID:36678193 |
|  | Chen J, Ngo C. Deep-based Ingredient Recognition for Cooking Recipe Retrieval. Proceedings of the 24th ACM international conference on Multimedia New York, NY, USA: Association for Computing Machinery; 2016. p. 32–41. doi: 10.1145/2964284.2964315 |
|  | Chen J, Zhu B, Ngo C-W, Chua T-S, Jiang Y-G. A study of multi-task and region-wise deep learning for food ingredient recognition. IEEE Trans Image Process IEEE; 2020;30:1514–1526. doi: https://doi.org/10.1109/TIP.2020.3045639 |
|  | Chen J, Bertrand S, Galy O, Raubenheimer D, Allman-Farinelli M, Caillaud C. The design and development of a food composition database for an electronic tool to assess food intake in new caledonian families. Nutrients MDPI; 2021;13(5):1668. doi: https://doi.org/10.3390/nu13051668 |
|  | Chi Y, Yu C, Qi X, Xu H. Knowledge management in healthcare sustainability: a smart healthy diet assistant in traditional Chinese medicine culture. Sustainability MDPI; 2018;10(11):4197. doi: https://doi.org/10.3390/su10114197 |
|  | Hinojosa-Nogueira D, Pérez-Burillo S, Navajas-Porras B, Ortiz-Viso B, de la Cueva SP, Lauria F, Fatouros A, Priftis KN, González-Vigil V, Rufián-Henares JÁ. Development of an unified food composition database for the european project “Stance4Health.” Nutrients MDPI; 2021;13(12):4206. doi: https://doi.org/10.3390/nu13124206 |
|  | Min W, Liu C, Xu L, Jiang S. Applications of knowledge graphs for food science and industry. Patterns Elsevier; 2022;3(5). doi: 10.1016/j.patter.2022.100484 |
|  | Richardson LC, Bazaco MC, Parker CC, Dewey-Mattia D, Golden N, Jones K, Klontz K, Travis C, Kufel JZ, Cole D. An updated scheme for categorizing foods implicated in foodborne disease outbreaks: a tri-agency collaboration. Foodborne Pathog Dis Mary Ann Liebert, Inc. 140 Huguenot Street, 3rd Floor New Rochelle, NY 10801 USA; 2017;14(12):701–710. doi: https://doi.org/10.1089/fpd.2017.2324 |
|  | Rothwell JA, Perez-Jimenez J, Neveu V, Medina-Remón A, M’Hiri N, García-Lobato P, Manach C, Knox C, Eisner R, Wishart DS, Scalbert A. Phenol-Explorer 3.0: a major update of the Phenol-Explorer database to incorporate data on the effects of food processing on polyphenol content. Database 2013 Jan 1;2013:bat070. doi: 10.1093/database/bat070 |
|  | Scarpa G, Berrang-Ford L, Bawajeeh AO, Twesigomwe S, Kakwangire P, Peters R, Beer S, Williams G, Zavaleta-Cortijo C, Namanya DB. Developing an online food composition database for an Indigenous population in south-western Uganda. Public Health Nutr Cambridge University Press; 2021;24(9):2455–2464. doi: 10.1017/S1368980021001397 |
|  | Tahir GA, Loo CK. A Comprehensive Survey of Image-Based Food Recognition and Volume Estimation Methods for Dietary Assessment. Healthcare Multidisciplinary Digital Publishing Institute; 2021 Dec;9(12):1676. doi: 10.3390/healthcare9121676 |
|  | Zeng Guo. Interpretation of Chinese Nutrition Society’s “Dietary Guidelines for Pregnant Women (2016).” Journal of Practical Obstetrics and Gynecology 2018;34(4):265–267. |
|  | Cui Guiyou. Some basic concepts in the science of cooking ingredients. Chinese Cooking Research 1998; (2): 26 to 30. |
|  | Li Xinhang. Establishment and Application In DietingPsychology of Chinese Food Image Library. Master’s thesis. Southwest University;​ 2018. Available from: https://kns.cnki.net/kcms2/article/abstract?v=ZOnxTxd1G4J-yfE1MdMn0qTMEtZpdOpYBKWvWnLbdHCh1CiZT5nBRQFMndeMLry-NpdJRK08qc x9BX7fQMATpt716ACdylGH74_MRQPZiX_tbDXexC-zX69JPFObRYzb5yukMW8ySqZUv5ej6jueQeVCzzNkvja5Q03apKp_jbXghbbNMtYT0-C826D9PVQTSn eun7Rn5Sk. |
|  | Liu Linhu. Ingredient Based Food Recognition. Master’s thesis. University of Chinese Academy of Sciences; 2020. Available from: 10.27824 /, dc nki. GZKDX. 2020.000020. |
|  | Min Weiqing, Liu Linhu, Liu Yuxin, Luo Mengjiang, Jiang Shuqiang. A Survey on Food Image Recognition. Chinese Journal of Computers 2022; 45 (3): 542-566. The doi: 10.11897 / SP., j. 1016.2022.00542. |
|  | Wang Haiyan, Zhang Miao, Liu Hulin, Chen Xiao. Chinese food image recognition method based on improved ResNet. Journal of Shaanxi University of Science & Technology 2022; 40(1): 154-160. doi: 10.19481/j.cnki.issn2096-398x.2022.01.023. |
|  | Wang Lu. A Healthy Diet Knowledge Q/A System based on Knowledge Graph. Master’s thesis. Lanzhou University; 2020. doi: 10.27204/d.cnki.glzhu.2020.001921. |
|  | Chinese Nutrition Society Dietary Guidelines revision Expert Committee for women and children dietary guidelines revision expert working group. Dietary Guidelines for pregnant women. Journal of Clinical Pediatrics 2016;34(11):877–880. |
|  | Chang QQ, Dang SN, Yan H, Zeng LX, Yang JM. Relationship between Chinese dietary guidelines compliance index for pregnant women and congenital heart disease in offspring. Zhonghua Liu Xing Bing Xue Za Zhi 2022 Dec 1;43(12):1979–1985. PMID:36572473 |
|  | Chen J, Ngo C. Deep-based Ingredient Recognition for Cooking Recipe Retrieval. Proceedings of the 24th ACM international conference on Multimedia New York, NY, USA: Association for Computing Machinery; 2016. p. 32–41. doi: 10.1145/2964284.2964315 |
| Books | Institute of Nutrition and Health, Chinese Center for Disease Control and Prevention. Chinese Food Composition Table (Sixth Edition) Volume 1. Beijing: Peking University Medical Press; 2018. Available from: https://book.douban.com/subject/30357861/ [accessed Sep 27, 2024]ISBN:978-7-5659-1699-1 |
|  | Institute of Nutrition and Health, Chinese Center for Disease Control and Prevention. Chinese Food Composition Table (Sixth Edition) Volume 2. Beijing: Peking University Medical Press; 2019. Available from: https://book.douban.com/subject/34869858/ [accessed Sep 27, 2024]ISBN:978-7-5659-1978-7 |
|  | Chinese Society of Nutrition. Dietary Guidelines for Chinese Residents (2022). Beijing: People’s Medical Publishing House; 2022. Available from: https://book.douban.com/subject/35885945/ [accessed Sep 27, 2024]ISBN:978-7-117-31404-6 |
|  | Chinese Society of Nutrition. Dietary Guidelines for Chinese Residents (2016). Beijing: People’s Medical Publishing House; 2016. Available from: https://book.douban.com/subject/26795949/ [accessed Sep 27, 2024]ISBN:978-7-117-22214-3 |
|  | Chinese Society of Nutrition. Report on the Scientific Research of Dietary Guidelines for Chinese Residents (2021). Beijing: People’s Medical Publishing House; 2022. Available from: https://book.douban.com/subject/35796207/ [accessed Sep 27, 2024]ISBN:978-7-117-32152-5 |
|  | Shao W. Culinary Technology (Third Edition). Beijing: Tourism Education Press; 2009. Available from: https://book.douban.com/subject/35779264/ [accessed Sep 27, 2024]ISBN:978-7-5637-4298-1 |
|  | Li N. Selected Home-cooked Dishes for Pregnancy. Beijing: China Light Industry Press; 2021. Available from: https://book.douban.com/subject/35646945/ [accessed Sep 27, 2024]ISBN:978-7-5184-3520-3 |
|  | Gan Z. 315 Examples of Nutritious Meals for Pregnancy Preparation and Pregnancy (Value Version). 2015. Available from: https://book.douban.com/subject/30754629/ [accessed Sep 27, 2024]ISBN:978-7-5537-4264-9 |
|  | Li N. Selected Home-cooked Dishes for Pregnancy Preparation. Beijing: China Light Industry Press; 2021. Available from: https://book.douban.com/subject/35695008/ [accessed Sep 27, 2024]ISBN:978-7-5184-3662-0 |
|  | Liu G. 40-Week Pregnancy Diet with the Right Food. Beijing: China Light Industry Press; 2021. Available from: https://book.douban.com/subject/35510919/ [accessed Sep 27, 2024]ISBN:978-7-5184-3439-8 |
|  | Sabatina. Saba Kitchen: Nutritious Recipes for Pregnancy. Beijing: China Light Industry Press; 2020. Available from: https://book.douban.com/subject/34950752/ [accessed Sep 27, 2024]ISBN:978-7-5184-2729-1 |
|  | Li N. Selected Home-cooked Dishes for Confinement. Beijing: China Light Industry Press; 2022. Available from: https://book.douban.com/subject/35732243/ [accessed Sep 27, 2024]ISBN:978-7-5184-3700-9 |
|  | Liu G. Have a Good 42-Day Confinement Meal. Beijing: China Light Industry Press; 2021. Available from: https://book.douban.com/subject/35510921/ [accessed Sep 27, 2024]ISBN:978-7-5184-3401-5 |
|  | Sabatina. Saba Kitchen: Nutritious Recipes for Confinement Period. Beijing: China Light Industry Press; 2020. Available from: https://book.douban.com/subject/35012492/ [accessed Sep 27, 2024]ISBN:978-7-5184-0774-3 |
| Knowledge graphs | FoodOn. Available from: https://foodon.org/ [accessed Sep 27, 2024] |
|  | Amith M, Onye C, Ledoux T, Xiong G, Tao C. The ontology of fast food facts: conceptualization of nutritional fast food data for consumers and semantic web applications. BMC Medical Informatics and Decision Making Springer Science and Business Media LLC; 2021 Nov;21(S7). doi: 10.1186/s12911-021-01636-1 |
| Internet | Xiachufang. Available from: https://www.xiachufang.com/ [accessed Sep 27, 2024] |
|  | Boohee. Available from: https://www.boohee.com/food [accessed Sep 27, 2024] |
|  | Bing Images. Bing. Available from: https://cn.bing.com/images/ [accessed Sep 27, 2024] |
|  | Baidu Image. Available from: https://image.baidu.com/ [accessed Sep 27, 2024] |
|  | Meishij. Available from: https://www.meishij.net/ [accessed Sep 27, 2024] |
